# Molecular Drivers of Chromophobe Renal Cell Carcinoma Revealed Through Genomic Analysis Using AACR Project GENIE

**DOI:** 10.3390/life15121909

**Published:** 2025-12-13

**Authors:** Abbi Gobel, Grace S. Saglimbeni, Eugene Manu, Nikhita Tandon, Tyson J Morris, Akaash Surendra, Beau Hsia, Peter T. Silberstein, Khalid Bashir, Abubakar Tauseef

**Affiliations:** 1 School of Life Sciences, Arizona State University, Tempe, AZ 85281, USA; abbigobel01@gmail.com (A.G.); asurend5@asu.edu (A.S.); 2 School of Medicine, Creighton University, Phoenix, AZ 85012, USA; gracesaglimbeni@creighton.edu (G.S.S.); tysonmorris@creighton.edu (T.J.M.);; 3 Hofstra College of Liberal Arts and Sciences, Hempstead, NY 11549, USA; eugenemanu4@gmail.com; 4 School of Biological Sciences, University of California, Davis, CA 95616, USA; nikhitatandon7@gmail.com; 5 School of Medicine, Creighton University, Omaha, NE 68178, USA; petersilberstein@creighton.edu (P.T.S.); khalid.bashir@commonspirit.org (K.B.)

**Keywords:** chromophobe renal cell carcinoma, non-clear renal cell carcinoma, renal cell carcinoma

## Abstract

Chromophobe renal cell carcinoma (chRCC) is a distinct subtype of non–clear cell renal cell carcinoma (ncRCC), arising from intercalated cells of the distal nephron collecting ducts. No standard treatments are specifically approved for chRCC, which is further hindered by lack of a universally accepted grading system. This study sought to find molecular drivers that may aid in the diagnosis or development of treatments for chRCC. A retrospective analysis of chRCC was conducted using data from the American Association for Cancer Research (AACR) Project Genomics Evidence Neoplasia Information Exchange (GENIE) repository, accessed through cBioPortal (version 17.0-public) on 21 July 2025. The study examined recurrent somatic mutations and assessed co-occurrence with Benjamini–Hochberg False Discovery Rate (FDR) correction. Additional analyses evaluated mutation by sex and race, with significance set at *p* < 0.05. The cohort included 180 tumor samples from 170 chRCC patients. Most patients were adults (*n* = 167, 98.2%) and White (*n* = 115, 67.6%). Recurrent alterations occurred in genes part of the p53, PI3K/mTOR, Hippo, and NOTCH signaling pathway. Exploratory demographic analyses identified isolated single-patient mutations in select genes across sex and race; however, these rare events are not interpretable as population-level differences. This study provides a comprehensive genomic profile of chRCC across multiple demographic categories.

## 1. Introduction

Chromophobe renal cell carcinoma (chRCC) is a rare malignant epithelial tumor of the kidney arising from intercalated cells of the distal nephron. Cancer development is driven by the interplay of genetic mutations and environmental exposures, which disrupt critical cellular processes including DNA repair, cell-cycle regulation, apoptosis, and tissue homeostasis, ultimately promoting uncontrolled proliferation and tumor formation [1]. The World Health Organization (WHO) classifies chRCC as a distinct entity within the category of “Oncocytic and Chromophobe Renal Tumors” [2]. Although some studies have proposed the existence of chRCC subtypes, the WHO does not currently endorse any formal variants due to the absence of meaningful prognostic differences [2]. Instead, the WHO recognizes two primary histologic patterns: “classic” and “eosinophilic” [2].

In the United States, chRCC accounts for approximately 3250 new cases annually, representing roughly 5% of all renal cell carcinomas (RCCs) [3]. Individuals with Birt Hogg Dube syndrome, family history of RCC, or a history of smoking or hypertension are at a higher predisposition to chRCC [3,4].

Clinically, chRCC is often asymptomatic at early stages, with patients manifesting symptoms such as flank pain or hematuria in later stages [3,5]. Patients with chRCC have a median survival of approximately 29 months, underscoring the unfavorable prognosis associated with advanced disease and the need for improved prognostic tools and therapeutic approaches [6].

Systemic therapy options for chRCC remain limited. Although VEGF-targeted tyrosine kinase inhibitors (TKIs), such as sunitinib and cabozantinib, have demonstrated modest activity in non-clear cell RCC, their efficacy in chRCC is markedly lower compared to clear cell RCC [5]. Similarly, immune checkpoint inhibitors have shown inconsistent response patterns in chRCC, reflecting the unique biology of this subtype [5]. As a result, treatment strategies for advanced chRCC remain challenging, underscoring the need for improved molecular characterization to guide precision therapeutic development.

This study represents the largest multi-institutional real-world genomic characterization of chRCC to date and provides expanded mutation frequency estimates, exploratory demographic analyses, and pathway-level interpretation across heterogeneous platforms. By integrating molecular and demographic data, this investigation aims to identify potential therapeutic targets and molecular drivers for the development of more precise and effective treatments for chRCC patients.

## 2. Materials and Methods

This was deemed exempt from institutional review board (IRB) oversight by Creighton University (Phoenix, AZ, USA) as it exclusively utilized de-identified, publicly accessible data from the American Association for Cancer Research (AACR) Project Genomics Evidence Neoplasia Information Exchange (GENIE) database. Clinical and genomic data were accessed via the cBioPortal platform (v17.0-public) on 21 July 2025, encompassing cases archived from 2017 onward. The AACR GENIE repository compiles genomic sequencing data from 19 international cancer centers and reflects considerable heterogeneity in sequencing methodologies, including whole-genome sequencing (WGS), whole-exome sequencing (WES), and targeted gene panels ranging from 50 to 555 genes. Because sequencing panels varied (50–555 genes) and varied across institutions, mutation absence may reflect non-coverage rather than true wild-type status. Rare germline variants may have also persisted as a result of the sequencing being tumor-only. Sequencing depth varied by platform: targeted panels achieved coverage exceeding 500×, WES had an average depth of ~150×, and WGS reached ~30× coverage. For chRCC, 99.4% of samples were sequenced using targeted gene panels, 0.6% with WES, and 0.0% with WGS. Regarding sample composition, approximately 70% of specimens in our chRCC cohort were derived from primary tumors and 99.5% of cases were tumor tissue samples, with a small proportion (0.5%) consisting of cfDNA. Matched tumor–normal information was not explicitly annotated, limiting the ability to exclude germline variants across the cohort.

Our study cohort consisted of patients with a confirmed pathological diagnosis of ChRCC, identified from a larger pool of renal cell carcinoma cases. Tumor samples were categorized as either primary (originating from the initial tumor site) or metastatic (from distant sites). Differences in gene-level mutation frequencies between primary and metastatic tumors were analyzed using a chi-squared test, comparing the proportion of mutated samples in each group. The dataset encompassed somatic mutation profiles, histological classifications, and clinical demographic information, including race, sex, and age. We also analyzed copy number alterations (CNAs), specifically targeting homozygous deletions and amplifications, and determined the frequency of recurrent events. Although panel designs varied by institution, most included core oncogenes and tumor suppressor genes such as *TP53*, *PTEN*, and *KMT2C*.

While each participating institution applied its own pipeline for somatic variant calling and annotation, all followed GENIE harmonization standards as defined by Genome NEXUS. Commonly used tools included GATK for variant detection and ANNOVAR for functional annotation, though specific software versions and parameter settings varied across sites. Despite these centralized harmonization efforts, some variability in bioinformatic processing likely persists both across and within contributing institutions. Clinical outcome and therapeutic response data are available for select cancer types within GENIE; however, treatment regimens were not recorded for chRCC.

All statistical computations were performed using R/RStudio (R Foundation for Statistical Computing, Boston, MA, USA, version 4.5.1), with *p*-values < 0.05 considered statistically significant. Continuous variables are expressed as mean ± standard deviation (SD), and categorical variables are presented as counts and percentages. Associations between categorical variables were analyzed using the chi-squared test. For continuous variables, data distribution was assessed to determine normality. Student’s *t*-test was applied for normally distributed data, while the Mann–Whitney U test was used for non-normally distributed variables. Multiple-testing correction was performed using the Benjamini–Hochberg false discovery rate method for analyses involving simultaneous comparisons.

Mutation data were obtained from GENIE’s harmonized MAF (mutation annotation format) files, which provide standardized gene- and protein-level annotations across contributing institutions. Only nonsynonymous somatic mutations (missense, frameshift, nonsense, and splice-site) were retained, with inclusion criteria of variant allele frequency (VAF) ≥ 5% and sequencing coverage ≥ 100×. Nonsynonymous somatic variants were included regardless of predicted pathogenicity due to variability in annotation across GENIE institutions. As such, some reported alterations may represent passenger mutations rather than drivers. Synonymous mutations, variants of uncertain significance, and genes without established clinical relevance were excluded. Structural variants were not assessed, and any samples with missing data were also removed from analysis. Only one tumor sample per patient was included for calculation of mutation frequencies. All mutation frequency calculations were performed at the patient level.

## 3. Results

### 3.1. chRCC Patient Demographics

A total of 180 tumor samples representing 170 unique patients with chRCC were included in the analysis. Patient characteristics are summarized in Table 1. The cohort consisted of 90 males (52.9%) and 78 females (45.9%). Nearly all patients were adults (*n* = 167), with only three pediatric cases identified. Ethnicity data showed that 120 patients (70.6%) were non-Hispanic/non-Spanish, 15 (8.8%) were Hispanic or Spanish, and 35 (20.6%) lacked recorded ethnicity. Regarding race, 115 patients (67.6%) were White, 8 (4.7%) were Asian, 6 (3.5%) were Black, 1 (0.6%) was Native American, 19 (11.2%) reported another racial background, and 21 (12.3%) had unreported racial information. Most tumors originated from primary sites (*n* = 124; 68.9%), with 45 samples (25.0%) collected from metastatic lesions and 11 (6.1%) without site annotation.

Among the metastatic samples with available site annotation, the most common metastatic sites included lung, liver, bone, and lymph nodes. Although limited by incomplete reporting, these data provide insight into metastatic patterns in chRCC. Sequencing methodologies for the cohort consisted primarily of targeted gene panels (99.4%), with a minority of samples sequenced by whole-exome sequencing (0.6%). No samples underwent whole-genome sequencing.

### 3.2. Spectrum of Recurrent Mutations

The mutational landscape of chRCC is summarized in Table 2. *p*-values from comparisons with expected cell counts below 5 were excluded due to insufficient statistical reliability. Panel coverage varied significantly by institution; therefore, absence of a reported mutation does not confirm wild-type status. As summarized in Table 2, alterations in *TP53* were most common (*n* = 92; 51.1%), followed by *PTEN* (*n* = 29; 16.1%). Less frequent but recurrent mutations were observed in *TERT* (*n* = 12; 6.7%), *RB1* (*n* = 10; 5.6%), *ATM* (*n* = 10; 5.6%), and *SETD2* (*n* = 10; 5.6%). Additional recurrently mutated genes included *NOTCH1* (*n* = 9; 5.0%), *KMT2D* (*n* = 8; 4.4%), *TET2* (*n* = 7; 3.9%), *TSC2* (*n* = 7; 3.9%), *ATRX* (*n* = 7; 3.9%), *FAT1* (*n* = 6; 3.3%), *ZFHX3* (*n* = 6; 3.3%), *APC* (*n* = 6; 3.3%), and *CHEK2* (*n* = 5; 2.8%).

#### Functional Relevance of *SETD2, PTEN, TP53,* and *TERT*

**Figure 1:**
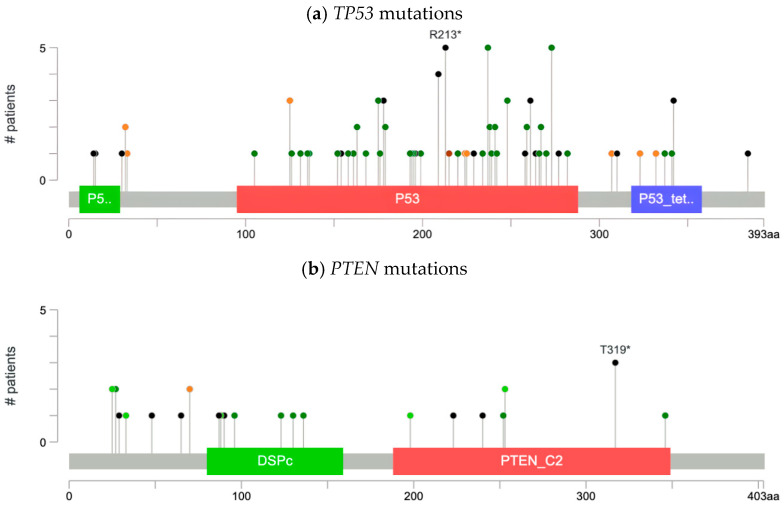

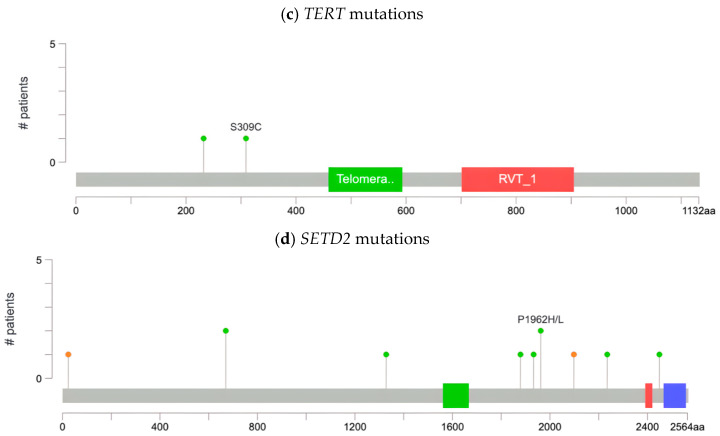
Lollipop plots of *SETD2*, *PTEN*, *TP53*, and *TERT*. Stem height represents the number of patients harboring mutations at each amino acid position within annotated protein domains. Color coding: mutation class is indicated by color—missense (green), truncating (black), in-frame (brown), and splice-site (orange), and fusion (purple)—with saturated colors denoting driver mutations and muted/desaturated color tones denoting variants of uncertain significance (VUS).

Our analysis characterized the types of mutations across frequently altered genes in our cohort: *SETD2*, *PTEN*, *TP53*, and *TERT*. As illustrated by the lollipop plot (Figure 1a), we identified 53 missense, 29 truncation, 1 in-frame, 13 splice, and 1 fusion mutation in *TP53* (*n* = 92), including recurrent hotspot alterations such as R213*, M237K, and R273H. R273H and M237K variants, which are both missense mutations, were found in the DNA-binding domain. R273H was found to increase cancer cell survival by erroneously activating the AKT signaling pathway. M237K, on the other hand, was identified by in vitro studies to render TP53 nonfunctional. R213*, a truncating mutation, was found to increase cancer cell proliferation and promote metastasis by aberrant localization to mitochondria. As a result, R213* is associated with poor prognosis in patients.

In *PTEN* (*n* = 29), we identified 9 missense mutations, 12 truncating mutations, 9 in-frame mutations, and 2 splice-site mutations, as illustrated in the lollipop plots (Figure 1b). *SETD2* (*n* = 10) and *TERT* (*n* = 12) mutations were dispersed throughout the gene, as illustrated by the lollipop plots (Figure 1c,d). For *SETD2*, we identified two splice mutations (Figure 1c). While for *TERT*, we identified 11 mutations of other types.

In addition to somatic mutations, we identified CNAs in 157 samples. Amplifications were observed in *TERT*, *YAP1*, and *BIRC3* (*n* = 3; 1.9% for all), while homozygous deletions were detected in *ERBB4* and *RB1* (*n* = 3; 1.9% for all).

### 3.3. Sex- and Race-Associated Variants

Sex-stratified analysis identified isolated single-patient mutations in several genes, including *MCM4*, *NUP214*, *SSX2*, and *TAF15* (Table 3). Because each of these variants occurred in only one individual, they are considered exploratory observations without interpretable biological significance.

When stratified by race, isolated single-patient mutations were also observed (Table 4). Examples include *PIK3C2B*, *STAT3*, *ERRFI1*, *TSC1*, *NCOR1*, *PPM1D*, and *ASXL2* in Black patients, and *FANCE*, *SLFN11*, *MLH3*, *CCN6*, *ALK*, and *CDKN1B* in Asian patients. *CHEK2* variants appeared in both Asian and White individuals. Because each event occurred in a single patient within very small racial subgroups (Black *n* = 6; Asian *n* = 8), these observations cannot be interpreted as recurring patterns or population-level associations.

Because all of these findings represent single mutation events within extremely small demographic subgroups, they should not be interpreted as biological enrichment or clinically meaningful differences. Instead, they serve as preliminary, hypothesis-generating signals that require validation in larger, more diverse datasets and cannot be generalized to broader populations.

### 3.4. Patterns of Co-Mutation and Exclusivity

Co-occurrence analysis demonstrated multiple significant gene–gene associations (Table 5). The most frequent involved *TP53* with *PTEN* (*n* = 19; 31.1%; *p* < 0.001). *TP53* also co-occurred with *RB1* (*n* = 8; 13.6%; *p* = 0.005) and *NOTCH1* (*n* = 6; 10.2%; *p* = 0.042). Additional co-mutation pairs included *PTEN* with *KMT2D* (*n* = 3; 13.6%; *p* = 0.004), *TSC2* with *FAT1* (*n* = 2; 40.0%; *p* = 0.001), *NOTCH1* with *KMT2D* (*n* = 2; 25.0%; *p* = 0.007), *SETD2* with *TSC2* (*n* = 2; 25.0%; *p* = 0.009), *NOTCH1* with *TSC2* (*n* = 2; 22.2%; *p* = 0.013), *TERT* with *RB1* (*n* = 3; 20.0%; *p* = 0.015), *RB1* with *TSC2* (*n* = 2; 18.2%; *p* = 0.022), *NOTCH1* with *ZFHX3* (*n* = 1; 33.3%; *p* = 0.030), *TSC2* with *ZFHX3* (*n* = 1; 33.3%; *p* = 0.030), *SETD2* with *NOTCH1* (*n* = 2; 18.2%; *p* = 0.031), and *PTEN* with *RB1* (*n* = 4; 14.8%; *p* = 0.037). Gene–gene associations were evaluated using log odds ratios. Due to low event counts, only *TP53* and *PTEN* demonstrated interpretable co-occurrence. Other associations lacked adequate support and are reported descriptively only.

Co-occurrence percentages were calculated by dividing the number of patients harboring both mutations by the number of patients with a mutation in either gene, defined as the sum of those with mutations in A only, B only, or both. Cases lacking alterations in both genes were excluded from the denominator.

## 4. Discussion

### 4.1. Subgroups and Mutational Landscape (Race, Gender, Age)

The aim of this investigation was to characterize the somatic mutational landscape of chromophobe renal cell carcinoma (chRCC) using the AACR Project GENIE repository. Our cohort consisted primarily of adults (*n* = 167, 98.2%), with only three pediatric patients. The scarcity of pediatric cases has hindered large-scale research in this population, leaving evidence-based treatment strategies poorly defined. The majority of patients in our cohort were White (67.6%), followed by Asian (4.7%) and Black (3.5%) patients. Sex stratification revealed a male-to-female distribution of 52.9% and 45.9%, respectively, with 1.2% unspecified. This pattern mirrors prior studies reporting balanced sex representation in chRCC [7]. Due to sample limitations, subgroup observations that could not undergo statistical correction should be considered preliminary and exploratory only.

Sex- and race-stratified analyses in this cohort identified several genes with isolated single-patient mutations. In the sex-based analysis, variants in *MCM4*, *NUP214*, *SSX2*, and *TAF15* were each detected in one female patient, with no corresponding cases in males. Similarly, the race-based analysis revealed single occurrences of mutations in genes such as *PIK3C2B*, *STAT3*, *ERRFI1*, *TSC1*, *NCOR1*, *PPM1D*, and *ASXL2* among Black patients, and *FANCE*, *SLFN11*, *MLH3*, *CCN6*, *ALK*, and *CDKN1B* among Asian patients, with *CHEK2* observed in individual Asian and White patients. However, because each of these findings reflects a single event within very small demographic subgroups (Black *n* = 6; Asian *n* = 8), they do not represent reproducible differences or reliable subgroup-specific patterns.

Given the extremely limited number of events and the absence of recurrent signals within these populations, these demographic observations should be interpreted solely as descriptive and exploratory. They do not support conclusions about biological, mechanistic, or prognostic differences based on sex or race. Instead, these isolated findings highlight areas that may warrant evaluation in larger, more diverse genomic datasets capable of supporting statistically meaningful subgroup analysis.

### 4.2. Commonly Mutated Genes and Altered Pathways

Genetic analysis of our cohort revealed substantial heterogeneity across multiple signaling pathways, including Hippo, *NOTCH*, chromosome stability, epigenetic regulation, and, most prominently, the p53 and PI3K/mTOR pathways. The most frequently altered genes were the tumor suppressors *TP53* (*n* = 92, 51.1%) and *PTEN* (*n* = 29, 16.1%), implicating the p53 and mTOR pathways in tumorigenesis. Compared with TCGA findings, which reported *TP53* mutation rates of ~32% and *PTEN* mutations of ~9%, our cohort demonstrated higher frequencies (51.1% and 16.1%, respectively). The higher mutation frequencies may also have been a result of differences in panel design, patient selection, or inclusion of variants of uncertain significance. Mutations in genes without known relevance to renal tumorigenesis (e.g., *SSX2*, *CCN6*, *ASXL2*) are likely incidental findings related to broad panel coverage rather than biologically meaningful drivers. In addition, single-occurrence mutations such as *SLFN11* or *ALK* may reflect artifacts of panel coverage or low *VAF* and should be interpreted with caution. Additional alterations included missense and splicing mutations in *TSC2* (*n* = 7, 3.9%), a negative regulator of the mTOR pathway, and missense variants in *CHEK2* and *ATM*, both critical regulators of the p53 pathway.

Deep deletions were identified in genes involved in cell differentiation (*ZFHX3*, *n* = 6, 3.3%; *NOTCH1*, *n* = 9, 5.0%) and cell cycle regulation (*RB1*, *n* = 10, 5.6%). Mutations affecting chromosomal stability were also prevalent, particularly within telomerase-maintenance alleles (*TERT*, *n* = 12, 6.7%; *ATRX*, *n* = 7, 3.9%), DNA damage response and repair genes (*ATM*, *n* = 10, 5.6%; *SETD2*, *n* = 10, 5.6%; *CHEK2*, *n* = 5, 2.8%), and epigenetic modulators (*KMT2D*, *n* = 8, 4.4%; *TET2*, *n* = 7, 3.9%). *SETD2* (*n* = 10, 5.6%) and *TERT* (*n* = 12, 6.7%) truncations also affected chromatin remodeling machinery. *SETD2* alterations consisted primarily of truncating variants consistent with loss of histone methyltransferase activity, while *TERT* promoter mutations reflect activation through upstream regulatory disruption. Mutations involving the Hippo pathway were identified in *FAT1* (*n* = 6, 3.3%) and *APC* (*n* = 6, 3.3%).

#### 4.2.1. TP53/ATM/CHEK2 p53 Pathway

Alterations in the p53 tumor suppressor pathway have been extensively documented in RCC, functioning both as early drivers of tumorigenesis and as contributors to tumor progression [8,9,10,11]. Within our cohort, we observed frequent mutations in *TP53* (*n* = 92, 51.1%), *ATM* (*n* = 10, 5.6%), and *CHEK2* (*n* = 5, 2.8%), suggesting that most alterations within the p53 pathway occur through disruption of its DNA damage response mechanism.

*ATM* and *CHEK2* are upstream activators of the p53 protein, encoded by *TP53*. In response to DNA damage, *ATM* activates *CHEK2*, which subsequently stabilizes p53 by preventing degradation through the MDM2–MDM4 protein complex [12,13]. Activated p53 then induces cell cycle arrest to facilitate DNA repair or triggers apoptosis when damage is irreparable [12].

These findings suggest that pharmacologic activation of the p53 pathway may represent a promising therapeutic strategy to restore DNA damage response signaling in chRCC. MDM2 inhibitors, such as APG-115, Nutlin-3a, and low dosage of actinomycin D, enhance p53 activity by preventing formation of the MDM2–MDM4 complex [14,15]. Additionally, multi-targeted kinase inhibitors sorafenib and sunitinib, which are both approved for clear cell RCC, have also been reported to increase p53 expression. Notably, prior research has shown that Nutlin-3a enhances the efficacy of sorafenib by further upregulating p53 [16]. Moreover, APR-246 (PRIMA-1MET), currently in phase III clinical trials, has demonstrated the ability to restore mutant p53 function while simultaneously upregulating p53 DNA damage response signaling [17].

#### 4.2.2. mTOR Pathway

Alterations in the mTOR pathway have been well documented in RCC and are implicated in tumor progression and metastasis [9,18,19]. The mTOR pathway serves as a central regulator of cell growth, proliferation, and survival [19]. In our cohort, approximately 20% of mutations are regulators of this pathway. Although mutations in *MTOR* itself, previously reported in chRCC, were not detected, we observed frequent alterations in *PTEN* (*n* = 29, 16.1%) and *TSC2* (*n* = 7, 3.9%) [17,20,21].

*PTEN* and *TSC2* act as tumor suppressors by negatively regulating *AKT*, a key activator of mTOR signaling [22]. Under normal conditions, *PTEN* dephosphorylates PIP3 to PIP2, thereby preventing AKT activation [22]. PIP3 functions as a membrane docking site that enables AKT phosphorylation by PDK1 and mTORC2 [22]. Loss of *PTEN* disrupts this regulation, leading to constitutive AKT activation and persistent stimulation of the mTOR pathway [22].

TSC1-TSC2 complex encodes a protein complex that represses mTOR [22]. *TSC2* encodes tuberin, a GTPase-activating protein that inhibits the small GTPase Rheb, one of the principal activators of mTOR [22]. Mutations in *TSC2*, or inhibition of *TSC2* function by excessive AKT activity, lead to persistent activation of the mTOR pathway [22]. Mutations in *TSC1* or *TSC2* are also associated with tuberous sclerosis complex (TSC), an autosomal dominant disorder characterized by benign tumors in the lungs, kidneys, or heart [22,23]. In TSC, hyperactivation of mTOR promotes degradation of insulin receptor substrates, reducing PI3K signaling, PIP3 formation, and AKT activation, thereby ultimately suppressing the mTOR pathway [22].

Therapeutically, mTOR inhibitors such as everolimus and temsirolimus are commonly used in RCC to target mTORC1 [24]. However, these agents may have limited efficacy in patients harboring concurrent *PTEN* and *TSC2* mutations due to the feedback loop between AKT and mTORC1 [25]. Inhibition of mTORC1 relieves suppression of AKT, resulting in increased AKT activity and continued upregulation of the mTOR pathway [25]. Consequently, AKT inhibitors may represent a more promising therapeutic strategy for patients with *PTEN* or *TSC2* mutations.

#### 4.2.3. Hippo Pathway

Mutations in the *FAT1* gene (3.3%) have been implicated in RCC tumor progression through disruption of the Hippo signaling pathway, which controls tissue growth by regulating cell cycle pathways [26]. In preclinical RCC models, the FAT1 protein functions as a tumor suppressor by acting as a cell-surface inhibitor of YAP1 [27]. Loss-of-function mutations in *FAT1* result in constitutive YAP1 activation, which subsequently stimulates the ERK/MAPK, PI3K/AKT, and RAS/RAF pathways, all of which play crucial roles in regulating cell differentiation [27]. These findings suggest that YAP1 inhibitors may represent an effective treatment strategy for chRCC patients with *FAT1* mutations.

#### 4.2.4. NOTCH Pathway

In RCC, dysregulation of the *NOTCH* signaling pathway contributes to tumorigenesis by promoting cell proliferation and inhibiting cell differentiation [28]. In our cohort, we identified alterations in *NOTCH1* (5%). Previous studies have reported mutations in other NOTCH receptors in chRCC; however, *NOTCH1* has been shown to exhibit particularly elevated expression in chRCC compared to other RCC subtypes [28,29,30]. In a cohort of 52 clear cell renal cell carcinoma patients, knockdown of *NOTCH1* was shown to induce apoptosis, suggesting that targeted inhibition of *NOTCH1* may represent a promising therapeutic approach for eliminating malignant cells [31].

### 4.3. Co-Occurrence Patterns and Functional Implications

In our chRCC cohort, we observed a high frequency of co-occurring mutations in *TP53* and *PTEN* (*n* = 19, 31.1%, *p* < 0.001), *TSC2* and *FAT1* (*n* = 2, 40.0%, *p* = 0.001), and *PTEN* and *KMT2D* (*n* = 3, 13.6%, *p* = 0.004). Although no mutually exclusive mutation pairs were identified, prior studies in RCC have reported recurrent alterations in *TP53* and *PTEN*, with chRCC exhibiting particularly high mutation rates in both genes [5,19,20,31]. Furthermore, *TP53* and *PTEN* mutations have been shown to accumulate during metastatic progression in chRCC, suggesting that their co-occurrence may serve as a potential biomarker of metastatic disease [9].

### 4.4. Limitations

This study has several limitations inherent to the AACR Project GENIE repository. First, demographic analyses are limited by extremely small subgroup sizes and should be interpreted with caution. Although one sample per patient was used for frequency calculations, potential institutional clustering effects may influence observed patterns. Second, the absence of treatment response or survival data precluded analysis of the association between therapy response and mutational profiles, as well as evaluation of treatment-induced genomic alterations driving tumor progression. Third, the repository’s sequencing data were aggregated from multiple institutional centers, which may introduce heterogeneity in the sequencing platforms used in the dataset. Fourth, the lack of longitudinal samples from individual patients restricted our ability to assess temporal dynamics of mutational patterns and their role in metastatic progression. This limitation was compounded by the integration of both primary and metastatic samples, regardless of chRCC subtype identity, into a single dataset. Fifth, because sequencing panels varied (50–555 genes) and varied across institutions, mutation absence may reflect non-coverage rather than true wild-type status. Rare germline variants may have also persisted as a result of the sequencing being tumor-only. Sixth, the absence of transcriptomic data prevented evaluation of how mutations influence gene expression or downstream pathway activity. This gap was particularly limiting for genes involved in epigenetic regulation, such as *ATRX* and *TET2*, and for exploring the contribution of miRNA to chRCC pathogenesis. Seventh, the lack of methylation data obstructed analysis of DNA methylation patterns and their role in tumor progression. Eighth, inclusion of multiple tumor samples from the same patient may have introduced bias; however, prior reports suggest that this does not significantly impact overall findings. Finally, the absence of immunohistochemistry data limited our ability to correlate mutation patterns with protein expression or assess immune-related biomarkers.

## 5. Conclusions

By leveraging a large, multi-institutional genomic dataset, this study provides a comprehensive characterization of the somatic mutational landscape of chRCC. These findings provide preliminary molecular insights that may be hypothesis-generating for future precision oncology studies, though they cannot inform clinical practice without functional or outcome validation. Moving forward, incorporation of multi-omics data, longitudinal sampling, and treatment information will be essential to contextualize mutation patterns, clarify their functional significance, and translate genomic discoveries into clinically actionable insights. Together, these efforts highlight the promise of developing personalized treatments tailored to chRCC to improve outcomes for patients with this rare and understudied subtype of RCC.

## Figures and Tables

**Table 1:** Patient demographics in chRCC.

Demographics	Category	*N* (%)
Sex	Male	90 (52.9)
	Female	78 (45.9)
Age category	Adult	167 (98.2)
	Pediatric	3 (1.8)
Ethnicity	Non-Hispanic	120 (70.6)
	Unknown/Not Collected	35 (20.6)
	Hispanic	15 (8.8)
Race	White	115 (67.6)
	Asian	8 (4.7)
	Black	6 (3.5)
	Native American	1 (0.6)
	Other	19 (11.2)
	Unknown/Not Collected	21 (12.3)
Sample Type	Primary	124 (68.9)
	Metastasis	45 (25.0)
	Not Collected	11 (6.1)

**Table 2:** Mutation frequency in chRCC.

Gene	*N* (%)
*TP53*	92 (51.1)
*PTEN*	29 (16.1)
*TERT*	12 (6.7)
*RB1*	10 (5.6)
*ATM*	10 (5.6)
*SETD2*	10 (5.6)
*NOTCH1*	9 (5.0)
*KMT2D*	8 (4.4)
*TET2*	7 (3.9)
*TSC2*	7 (3.9)
*ATRX*	7 (3.9)
*FAT1*	6 (3.3)
*ZFHX3*	6 (3.3)
*APC*	6 (3.3)
*CHEK2*	5 (2.8)

**Table 3:** Somatic mutation observed by sex.

Gene	Female, *N* (%)	Male, *N* (%)
*MCM4*	1 (100.0)	0 (0.0)
*NUP214*	1 (100.0)	0 (0.0)
*SSX2*	1 (100.0)	0 (0.0)
*TAF15*	1 (100.0)	0 (0.0)

**Table 4:** Somatic mutation observed by race.

Gene (Chi-Squared)	Asian, *N* (%)	Black, *N* (%)	White, *N* (%)
*PIK3C2B*	0 (0.0)	1 (100.0)	0 (0.0)
*STAT3*	0 (0.0)	1 (20.0)	0 (0.0)
*ERRFI1*	0 (0.0)	1 (25.0)	0 (0.0)
*FANCE*	1 (50.0)	0 (0.0)	0 (0.0)
*TSC1*	0 (0.0)	1 (16.7)	0 (0.0)
*SLFN11*	1 (50.0)	0 (0.0)	0 (0.0)
*NCOR1*	0 (0.0)	1 (20.0)	0 (0.0)
*PPM1D*	0 (0.0)	1 (20.0)	0 (0.0)
*ASXL2*	0 (0.0)	1 (20.0)	0 (0.0)
*ALK*	1 (12.5)	0 (0.0)	0 (0.0)
*CDKN1B*	1 (12.5)	0 (0.0)	0 (0.0)
*CHEK2*	2 (25.0)	0 (0.0)	2 (1.8)
*MLH3*	1 (50.0)	0 (0.0)	0 (0.0)
*CCN6*	1 (50.0)	0 (0.0)	0 (0.0)

**Table 5:** Significant co-occurring mutations in chRCC.

A	B	*N* (%)	*p* Value
*TP53*	*PTEN*	19 (31.1)	*p* < 0.001
*TSC2*	*FAT1*	2 (40.0)	*p* = 0.001
*PTEN*	*KMT2D*	3 (13.6)	*p* = 0.004
*TP53*	*RB1*	8 (13.6)	*p* = 0.005
*NOTCH1*	*KMT2D*	2 (25.0)	*p* = 0.007
*SETD2*	*TSC2*	2 (25.0)	*p* = 0.009
*NOTCH1*	*TSC2*	2 (22.2)	*p* = 0.013
*TERT*	*RB1*	3 (20.0)	*p* = 0.015
*RB1*	*TSC2*	2 (18.2)	*p* = 0.022
*NOTCH1*	*ZFHX3*	1 (33.3)	*p* = 0.030
*TSC2*	*ZFHX3*	1 (33.3)	*p* = 0.030
*SETD2*	*NOTCH1*	2 (18.2)	*p* = 0.031
*PTEN*	*RB1*	4 (14.8)	*p* = 0.037
*TP53*	*NOTCH1*	6 (10.2)	*p* = 0.042

## Data Availability

The data presented in this study are available from the AACR GENIE Database at https://genie.cbioportal.org/ (accessed on 21 July 2025).

## Abbreviations

The following abbreviations are used in this manuscript: AACR, American Association for Cancer Research; *AKT*, Protein Kinase B; *ALK*, Anaplastic Lymphoma Kinase; *ATM*, Ataxia Telangiectasia Mutated; *ATR*, Ataxia Telangiectasia and Rad3-related; *BRAF*, v-Raf Murine Sarcoma Viral Oncogene Homolog B; *CDKN2A*, Cyclin Dependent Kinase Inhibitor 2A; chRCC, Chromophobe Renal Cell Carcinoma; CNA, Copy Number Alteration; COSMIC, Catalogue of Somatic Mutations in Cancer; *EGFR*, Epidermal Growth Factor Receptor; *ERBB2*, Erb-B2 Receptor Tyrosine Kinase 2; *FLCN*, Folliculin; GENE, Genomics Evidence Neoplasia Information Exchange; GENIE, Genomics Evidence Neoplasia Information Exchange; indel, Insertion or Deletion; *KEAP1*, Kelch-like ECH-associated Protein 1; *mTOR*, Mechanistic Target of Rapamycin; ncRCC, Non–Clear Cell Renal Cell Carcinoma; NGS, Next-Generation Sequencing; *NRAS*, Neuroblastoma RAS Viral Oncogene Homolog; *PI3K*, Phosphoinositide 3-Kinase; *PTEN*, Phosphatase and Tensin Homolog; RCC, Renal Cell Carcinoma; *RHEB*, Ras Homolog Enriched in Brain; *ROS1*, ROS Proto-Oncogene 1; RTK, Receptor Tyrosine Kinase; *SDH*, Succinate Dehydrogenase; SNV, Single Nucleotide Variant; TCGA, The Cancer Genome Atlas; *TSC1*, Tuberous Sclerosis Complex 1; *TSC2*, Tuberous Sclerosis Complex 2; *VHL*, Von Hippel–Lindau; VUS, Variants of Uncertain Significance.
